# Tumor necrosis factor-α antagonist diminishes osteocytic RANKL and sclerostin expression in diabetes rats with periodontitis

**DOI:** 10.1371/journal.pone.0189702

**Published:** 2017-12-14

**Authors:** Ji-Hye Kim, Ae Ri Kim, Yun Hui Choi, Sungil Jang, Gye-Hyeong Woo, Jeong-Heon Cha, Eun-Jung Bak, Yun-Jung Yoo

**Affiliations:** 1 Department of Oral Biology, Yonsei University College of Dentistry, Seoul, Republic of Korea; 2 Department of Applied Life Science, The Graduate School, Yonsei University, Seoul, Republic of Korea; 3 BK21 PLUS Project, Yonsei University College of Dentistry, Seoul, Republic of Korea; 4 Department of Clinical Laboratory Science, Semyung University, Jecheon, Republic of Korea; Universite de Nantes, FRANCE

## Abstract

Type 1 diabetes with periodontitis shows elevated TNF-α expression. Tumor necrosis factor (TNF)-α stimulates the expression of receptor activator of nuclear factor-κB ligand (RANKL) and sclerostin. The objective of this study was to determine the effect of TNF-α expression of osteocytic RANKL and sclerostin in type 1 diabetes rats with periodontitis using infliximab (IFX), a TNF-α antagonist. Rats were divided into two timepoint groups: day 3 and day 20. Each timepoint group was then divided into four subgroups: 1) control (C, n = 6 for each time point); 2) periodontitis (P, n = 6 for each time point); 3) diabetes with periodontitis (DP, n = 8 for each time point); and 4) diabetes with periodontitis treated with IFX (DP+IFX, n = 8 for each time point). To induce type 1 diabetes, rats were injected with streptozotocin (50 mg/kg dissolved in 0.1 M citrate buffer). Periodontitis was then induced by ligature of the mandibular first molars at day 7 after STZ injection (day 0). IFX was administered once for the 3 day group (on day 0) and twice for the 20 day group (on days 7 and 14). The DP group showed greater alveolar bone loss than the P group on day 20 (*P* = 0.020). On day 3, higher osteoclast formation and RANKL-positive osteocytes in P group (P = 0.000 and P = 0.011, respectively) and DP group (P = 0.006 and P = 0.017, respectively) than those in C group were observed. However, there was no significant difference in osteoclast formation or RANKL-positive osteocytes between P and DP groups. The DP+IFX group exhibited lower alveolar bone loss (*P* = 0.041), osteoclast formation (*P* = 0.019), and RANKL-positive osteocytes (*P* = 0.009) than that of the DP group. On day 20, DP group showed a lower osteoid area (*P* = 0.001) and more sclerostin-positive osteocytes (*P* = 0.000) than P group. On days 3 and 20, the DP+IFX group showed more osteoid area (*P* = 0.048 and 0.040, respectively) but lower sclerostin-positive osteocytes (both *P* = 0.000) than DP group. Taken together, these results suggest that TNF-α antagonist can diminish osteocytic RANKL/sclerostin expression and osteoclast formation, eventually recovering osteoid formation. Therefore, TNF-α might mediate alveolar bone loss via inducing expression of osteocytic RANKL and sclerostin in type 1 diabetes rats with periodontitis.

## Introduction

Bone loss is determined by the degree of bone resorption and bone formation. Many factors can affect bone resorption and formation, including hormone levels, aging, and innervation [1–3]. Among various factors, receptor activator of nuclear factor-κB (RANKL) and sclerostin are known to affect bone resorption and formation, respectively. RANKL induces osteoclast formation via binding to RANK on osteoclast precursors [3]. RANKL is expressed in various cells, including osteoblasts, periodontal ligament cells, lymphocytes, and osteocytes [4–8]. Sclerostin regulates bone formation by interrupting Wnt signaling [9]. It binds to low-density lipoprotein receptor-related proteins 5 and 6 on the cell membrane of osteoblasts and inhibits canonical Wnt/β-catenin signaling, reducing osteoblastic bone formation. Sclerostin is expressed in osteocytes. In type 1 diabetes, bone resorption and bone formation are imbalanced and bone microstructure is altered, leading to bone fragility. Among complicated mechanisms of bone fragility, insulin deficiency with inflammation and glucose toxicity are contributing factors [10–12].

Periodontal disease is an inflammatory disease of periodontal tissues. It is characterized by alveolar bone loss. Patients with chronic periodontitis show higher RANKL levels in periodontal tissues than healthy individuals [13]. RANKL inhibition via osteoprotegerin, a RANKL inhibitor, blocks alveolar bone loss in rats with periodontitis [14]. Patients with chronic periodontitis have higher sclerostin levels in gingival tissues and serum than non-periodontitis individuals, suggesting a possible role of sclerostin in periodontal tissues [15]. In addition, sclerostin levels in gingival crevicular fluid are higher in patients with chronic periodontitis than those in healthy individuals. However, these levels are decreased after non-surgical periodontal treatment, suggesting that regulating sclerostin levels might be used as a new therapeutic strategy to treat periodontal disease [16]. Furthermore, wild type mice with periodontitis exhibit significant bone resorption compared to sclerostin knockout mice with periodontitis [17]. We have previously found that osteocytic sclerostin expression is inversely related to osteoid formation in rats with periodontitis [5]. Taken together, these findings suggest that RANKL and sclerostin expression is involved in alveolar bone loss in periodontitis.

Type 1 diabetes is a risk factor for periodontitis. Periodontitis is severe in type 1 diabetes patients [18–21]. It has been reported that streptozotocin (STZ)-induced type 1 diabetes rats with periodontitis exhibit more alveolar bone loss with less bone formation compared to rats with periodontitis only [22]. Interestingly, the low bone formation in type 1 diabetes rats with periodontitis is correlated with high osteocytic sclerostin expression, suggesting that bone formation might be suppressed in type 1 diabetes with periodontitis via high osteocytic sclerostin expression.

Tumor necrosis factor (TNF)-α is a pro-inflammatory cytokine expressed in periodontitis [15, 23, 24]. Patients with periodontitis and type 1 diabetes have higher expression levels of TNF-α than patients with periodontitis [25]. Similar finding has been reported in rats with both periodontitis and type 1 diabetes [26]. TNF-α induces RANKL expression in osteoblasts, periodontal ligament fibroblasts, and MLO-Y4, a murine osteocyte-like cell line [6, 27, 28]. It also induces sclerostin expression in MLO-Y4 cells [6, 29]. However, whether TNF-α stimulates osteocytic RANKL and sclerostin expressions in diabetes with periodontitis is currently unclear. Therefore, the objective of this study was to determine the effect of infliximab (IFX), a TNF-α antagonist, on osteocytic RANKL and sclerostin expression in STZ-induced type 1 diabetes rats with periodontitis.

## Materials and methods

### Animals

Six-week male inbred F344 rats were purchased from Orient Bio Inc. (Seongnam, Korea) and adapted for one week. These rats were housed in individual ventilated cages in groups of 3–4 animals per cage. They were provided access to fed standard rat chow (Orient-Bio Inc.) and water *ad libitum*. The animals were maintained in a temperature-controlled room (22°C) and 50 ± 10% humidity on a 12-hour light-dark cycle (light on 8:00–20:00). This study was carried out in strict accordance with the recommendations in the Guide for the Care and Use of Laboratory Animals of the National Institutes of Health. The animal protocols were approved by the institutional Animal Care and Use Committee of Yonsei University (approval number: 2014–0393). All efforts were made to minimize suffering.

### Study design

Using G*Power 3.1.9.2 software [30], it was predicted that at least 16 animals at each time point (n = 4 per group, four groups) would be needed with effect size of 1.12, 80% power, and alpha level at 0.05 in one-way analysis of variance (ANOVA). The experimental scheme is shown in S1A Fig. Fifty-six rats were divided into two timepoint groups: 3 day group (n = 28) and 20 day group (n = 28). Each timepoint group was then divided into the following 4 subgroups as follows: 1) untreated control (C, n = 6 at each time point); 2) periodontitis (P, n = 6 at each time point); 3) diabetes with periodontitis (DP, n = 8 at each time point), and 4) diabetes with periodontitis treated with IFX (DP+IFX, n = 8 at each time point) groups. We allocated two additional rats for DP and DP+IFX subgroups that underwent induction of diabetes. After fasting for 16 h, type 1 diabetes was induced by intravenous administration of STZ (Sigma-Aldrich, St Louis, MO, USA; 50 mg/kg dissolved in 0.1 M citrate buffer) under inhalation anaesthesia with 2% isoflurane in oxygen (2l/min) for 2 min. Rats in non-diabetes groups were injected with citrate buffer alone. At one week after administration of STZ or citrate buffer (day 0), rats were anesthetized intraperitoneally with zoletil 50 (30 mg/kg, Virbac, Carros, France) and rompun (10 mg/kg, Bayer Korea, Ansan, Korea). Periodontitis was induced by bilateral ligature of the mandibular first molars using dental floss (Procter & Gamble, Cincinnati, OH, USA). IFX (5 mg/kg, Janssen Biologics B. V., Leiden, Netherlands) was administrated intraperitoneally once for the 3 day group (on day 0) and twice for the 20 day group (on days 7 and 14). Each group was then sacrificed on either day 3 or day 20 after ligature using CO_2_ gas for reducing animal suffering.

Dental floss location and body weight were checked during the experiment periods (S1B Fig). Right prior to sacrifice, blood glucose levels were measured using an Accu-Check active system (Roche, Mannheim, Germany) after overnight fasting in accordance with the manufacturer’s instructions (S1C Fig).

### Histological procedures

Upon sacrifice, mandibles were extracted. After fixation with 10% neutral buffered formalin for two days, the mandibles were decalcified with 10% EDTA for two months. After tissue processing procedures (dehydration, clearing, and infiltration), they were embedded in paraffin, cut into serial 4 μm thick sagittal sections, and placed on silane-coated glass slides (Thermo Fisher Scientific, Waltham, MA, USA). Sections were selected based on clear appearance of dental pulp of mesial and distal roots of first molars.

### Histomorphological analysis to evaluate alveolar bone loss and formation

To quantify alveolar bone loss, the distance from the cementoenamel junction (CEJ) to the alveolar bone crest (ABC) in the distal area of the first molar was measured under 10× objective in hematoxylin and eosin (H&E) stained sections. To quantify new bone formation, the osteoid area in the distal alveolar bone of the first molar was measured under 20× objective according to previous studies [5]. The new osteoid was considered as unmineralized bone matrix (light pink color) between osteoblasts which are cuboidal bone-lining cells and mineralized bone surface stained as pink color in H&E sections. Images were taken using an Olympus microscope system (CKX41, Tokyo, Japan) and analyzed using ImagePro software (Media Cybernetics, Silver Spring, MD, USA). Measurement of osteoid formation was carried out in a region of interest (ROI) that extended 0.5 mm from the ABC in the distal alveolar bone of the first molar. The individual who performed the analysis was blinded to experimental conditions. Measurements of CEJ-ABC distance and osteoid area were conducted for all rats from each time point.

### Immunohistochemical analysis

Following deparaffinization in xylene and rehydration using ethanol, slide sections were immersed in 3% hydrogen peroxide for 20 min. Sections were incubated with trypsin (Thermo Fisher Scientific, Waltham, MA, USA) for antigen retrieval. ImmPRESS kits (MP-7405; Vector Laboratories, Burlingame, CA, USA) were used for immunohistochemistry staining according to the manufacturer’s instructions. Sections were blocked with normal horse blocking reagent (provided by ImmPRESS kits) for 20 min. To identify osteoclast formation, sections were immunostained with goat polyclonal anti-cathepsin K antibody (1:75; sc-6507; Santa Cruz Biotechnology Inc, Santa Cruz, CA, USA) at 4°C overnight. To measure RANKL and sclerostin expression in osteocytes, sections were immunostained with goat polyclonal anti‐RANKL antibody (1:500; sc-7628; Santa Cruz Biotechnology Inc.) and goat polyclonal anti‐sclerostin antibody (1:300; AF1589; R&D Systems, Minneapolis, MN, USA), respectively, at 4°C overnight. These section were incubated with secondary antibody (provided by ImmPRESS kits) followed by incubation with peroxidase-labeled streptavidin (Abcam, Cambridge, UK) for 20 min. Color was developed using substrate-chromogen (Dako, Carpinteria, CA, USA) and counterstained with methyl green (Trevigen, Gaithersburg, MD, USA). Primary antibodies were omitted as negative controls. Sections used as negative control did not display any specific immunoreactivity. Images were taken with an Olympus microscope system using 40× objective and analyzed with ImagePro software. Measurements of cathepsin K-positive multinucleated cells and RANKL- and sclerostin-positive osteocytes were carried out in a ROI that extended 0.5 mm from the ABC in the distal alveolar bone of the first molar. The number of cathepsin K-positive multinucleated cells was counted along the alveolar bone surface in the RIO and calculated per millimeter length of alveolar bone surface. The number of RANKL- and sclerostin-positive osteocytes was divided by the number of total osteocytes to calculate a percentage. The individual who performed the analysis was blinded to the experimental conditions. Measurements of cathepsin K-, RANKL- and sclerostin-positive cells were conducted for all rats from each time point.

### Real-time polymerase chain reaction

Alveolar bones and gingival tissues of rat first molars were collected from half number of each group at each time point (C, n = 3; P, n = 3; DP, n = 4; DP+IFX, n = 4 at each time point). Total RNA was extracted from alveolar bones and gingival tissues using TRIzol reagent (Invitrogen, Carlsbad, CA, USA) in accordance with the manufacturer’s instructions. One μg of total RNA was converted to complementary DNA (cDNA) using RT-PCR PreMix Kit (Bioneer, Seoul, Korea) according to the manufacturer’s instructions. Quantitative real-time PCR was conducted using SYBR Green PCR Master Mix (Applied Biosystems, Warrington, UK) and a MyiQ2 Two-Color Real-Time PCR Detection System (Bio-Rad, Hercules, CA, USA). Specific real-time PCR primers for IL-1β, sclerostin, and GAPDH were listed in S1 Table. Relative gene expression quantification was normalized to GAPDH mRNA expression using 2^-∆∆^ Ct analysis method. We performed melting curve analyses to verify amplification specificity and the absence of primer dimers.

### Statistical analyses

All values are presented as mean ± standard error (SE). Data were evaluated by ANOVA followed by Scheffe’s test. Statistical significance was determined using SPSS software (IBM, Chicago, IL, USA). A *P* value of less than 0.05 was considered statistically significant.

## Results

### Alveolar bone loss

**Fig 1:**
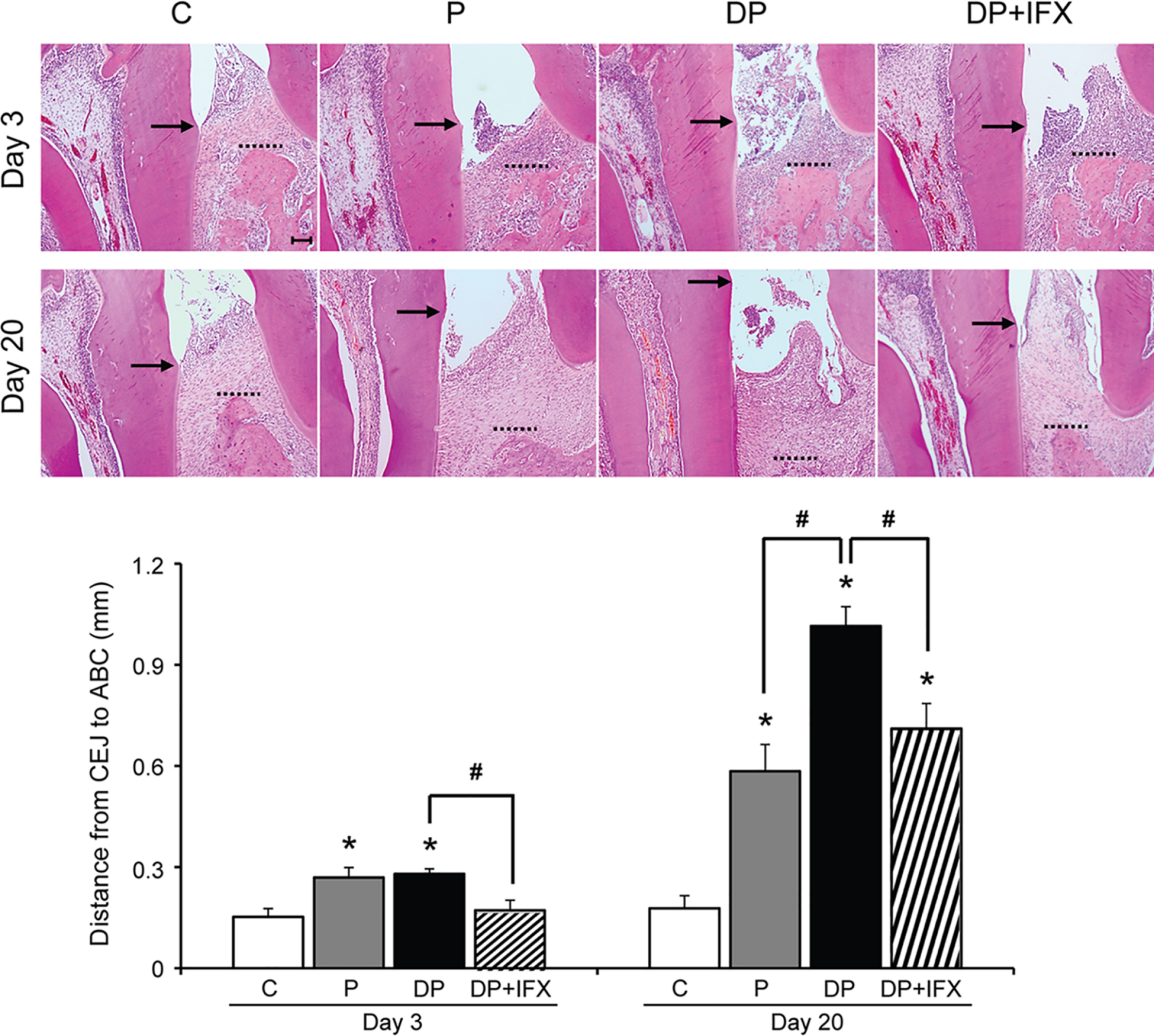
Alveolar bone loss. Representative images of the distance from CEJ to ABC in each group. Black arrows and dotted lines represent the CEJ and ABC, respectively (upper panels, H&E stain, scale bar = 100 μm). Measurements of the distance from CEJ to ABC in each group (lower panel, C, n = 6; P, n = 6; DP, n = 8; DP+IFX, n = 8 at each time point). Data are presented as mean ± SE. Data were evaluated by one-way analysis of variance (ANOVA) followed by Scheffe’s test. A *P* value of less than 0.05 was considered statistically significant. * *P* < 0.05 compared to C group; # *P* < 0.05; C: control group; P: periodontitis group; DP: diabetes with periodontitis group; DP+IFX: diabetes with periodontitis treated with infliximab group.

To observe the effect of IFX on alveolar bone loss in type 1 diabetes with periodontitis, we measured the distance of CEJ (black arrow) to ABC (dotted line) in the distal area (Fig 1). On day 3, distance from CEJ to ABC in the P or DP groups was longer than that in the C group while that in DP+IFX group was shorter than that in the DP group. On day 20, the distance from CEJ to ABC in the DP group was longer than that in the P group while that in the DP+IFX group was shorter than that in the DP group.

### Osteoclast formation

**Fig 2:**
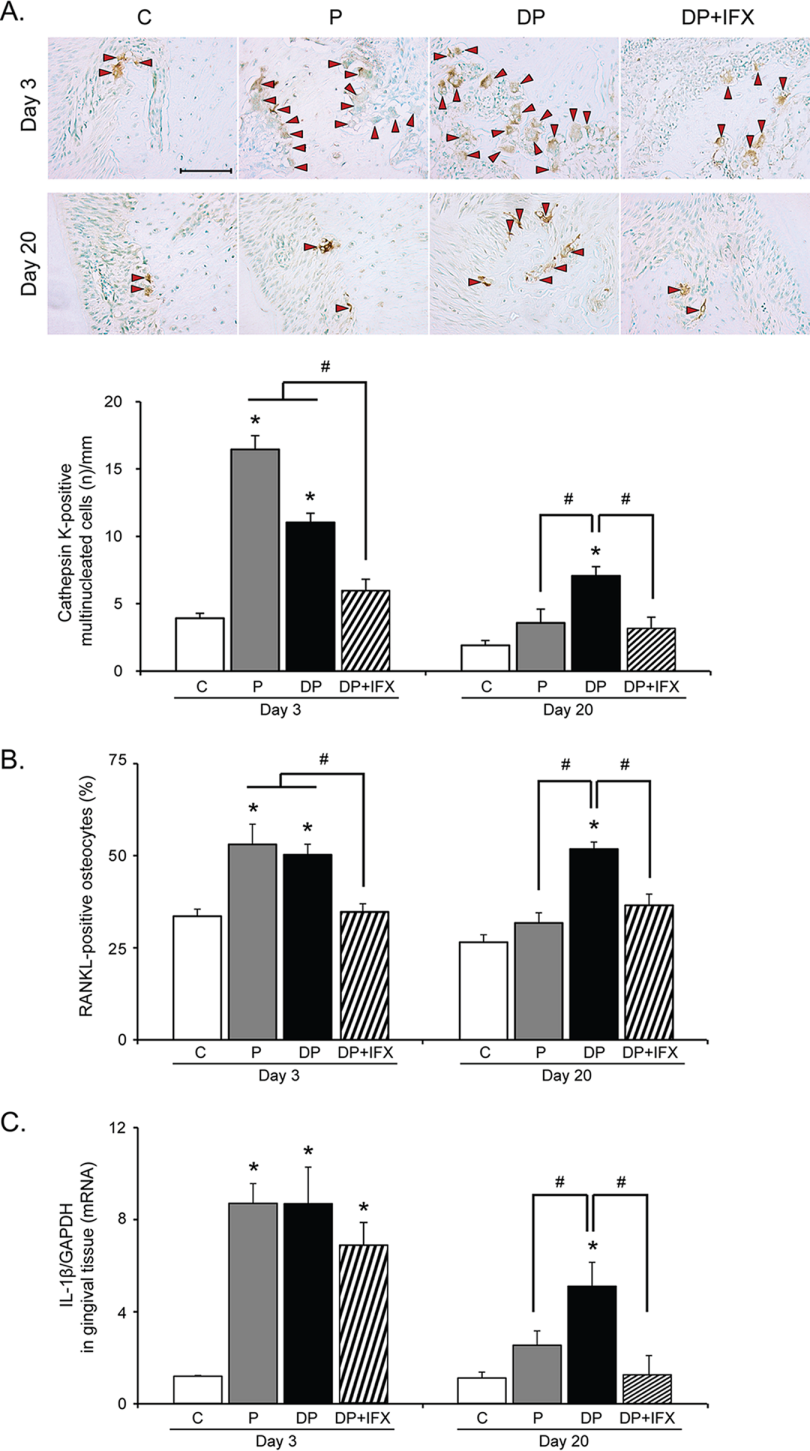
Cathepsin K-positive osteoclasts and RANKL-positive osteocytes in alveolar bone and IL-1β mRNA expression levels in gingival tissues. **(A)** Representative images of the number of cathepsin K-positive multinucleated cells in each group. Red arrowheads represent cathepsin K-positive multinucleated cells (upper panels, scale bar = 100 μm). Quantification of the number of cathepsin K-positive multinucleated cells in each group (lower panel, C, n = 6; P, n = 6; DP, n = 8; DP+IFX, n = 8 at each time point). **(B)** Percentage of RANKL-positive osteocytes in each group (C, n = 6; P, n = 6; DP, n = 8; DP+IFX, n = 8 at each time point). **(C)** IL-1β mRNA expression levels in gingival tissues of each group (C, n = 3; P, n = 3; DP, n = 4; DP+IFX, n = 4 at each time point). Data are presented as mean ± SE. Data were evaluated by one-way analysis of variance (ANOVA) followed by Scheffe’s test. A *P* value of less than 0.05 was considered statistically significant. * *P* < 0.05 compared to C group; # *P* < 0.05; n = number.

To evaluate the effect of IFX on osteoclast formation in type 1 diabetes with periodontitis, we counted cathepsin K-positive multinucleated cells in the distal area (Fig 2A). On day 3, the number of cathepsin K-positive cells in P or DP group was significantly higher than that in the C group. However, the number was not significantly different between P and DP groups. The number of cathepsin K-positive cells in DP+IFX group was lower than that in the DP or P group. On day 20, the number of cathepsin K-positive cells in DP group was higher than that in C, P, or DP+IFX group. There was no significant difference in the number of cathepsin K-positive cells among C, P, and DP+IFX groups.

### RANKL expression in osteocytes

To assess the effect of IFX on RANKL expression in type 1 diabetes with periodontitis, we counted the number of RANKL-positive osteocytes in the distal area (Fig 2B). On day 3, the number of RANKL-positive osteocytes in P or DP group was higher than that in the C group while that number in DP+IFX group was markedly lower than that in DP or P group. Interestingly, a high number of RANKL-positive osteocytes in the DP group was maintained on day 20 whereas DP+IFX and P groups exhibited lower numbers of RANKL-positive osteocytes compared to DP group. It is known that TNF-α stimulates interleukin (IL)-1β proinflammatory cytokine [24, 31, 32]. To determine whether IFX affected expression of IL-1β, IL-1β mRNA expression levels in the gingival tissues of first molars were measured (Fig 2C). On day 3, IL-1β mRNA expression levels in P, DP, and DP+IFX groups were higher than those in the C group. On day 20, we observed higher IL-1β mRNA expression in DP group than that in C or P group. However, IL-1β mRNA expression in DP+IFX group was lower than that in DP group.

### Bone formation in alveolar bone

**Fig 3:**
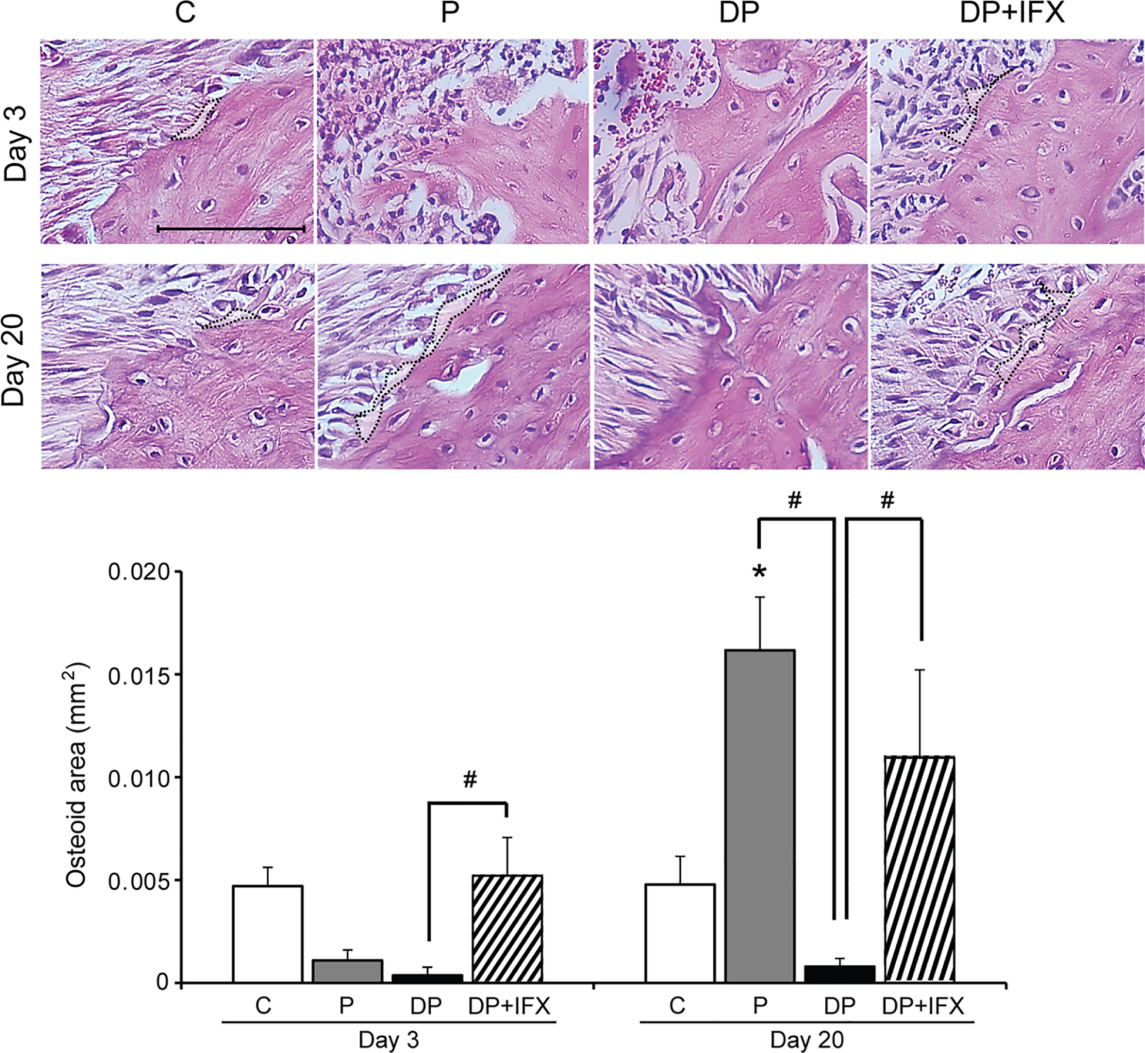
Osteoid area in alveolar bone. Representative images of osteoid area in each group. Black dotted lines indicate the osteoid area (upper panels, H&E stain, scale bar = 100 μm). Quantification of osteoid area in each group (lower panel, C, n = 6; P, n = 6; DP, n = 8; DP+IFX, n = 8 at each time point). Data are presented as mean ± SE. Data were evaluated by one-way analysis of variance (ANOVA) followed by Scheffe’s test. A *P* value of less than 0.05 was considered statistically significant. * *P* < 0.05 compared to C group; # *P* < 0.05.

To investigate the effect of IFX on bone formation in type 1 diabetes with periodontitis, the osteoid area was measured in the distal area (Fig 3). On day 3, the osteoid area in DP+IFX group was larger than that in DP group. On day 20, the osteoid area in P group was greater than that in C or DP group. Additionally, the osteoid area in DP+IFX group was much larger than that in DP group.

### Sclerostin expression in osteocytes

**Fig 4:**
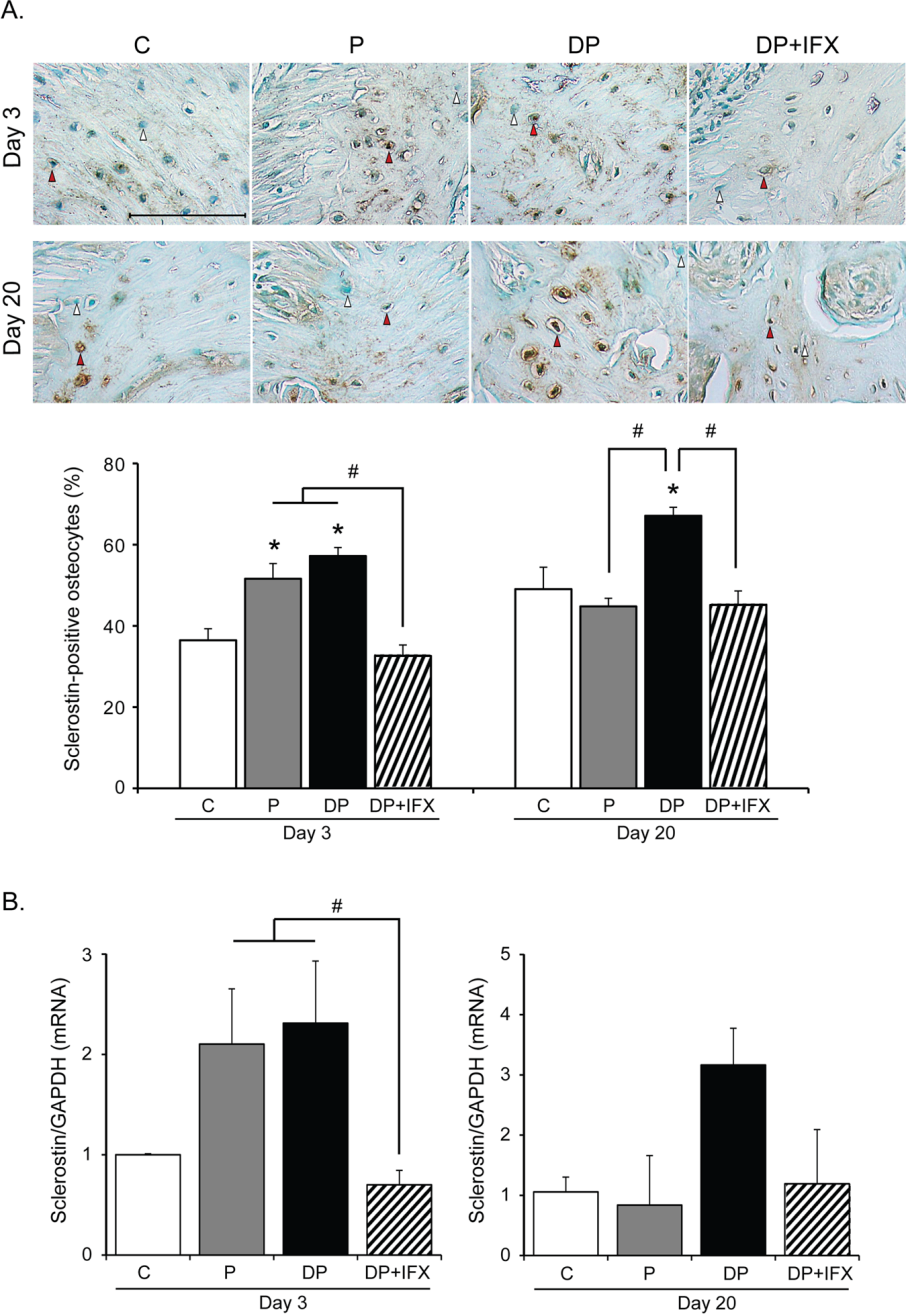
Sclerostin expression in osteocytes of alveolar bone. **(A)** Representative images of sclerostin-positive osteocytes in each group. White and red arrowheads indicate representative sclerostin-negative and -positive osteocytes, respectively (upper panels, immunohistochemistry stain, scale bar = 100 μm). Percentage of sclerostin-positive osteocytes in each group (lower panel, n = 6; P, n = 6; DP, n = 8; DP+IFX, n = 8 at each time point). **(B)** Sclerostin mRNA expression in each group (C, n = 3; P, n = 3; DP, n = 4; DP+IFX, n = 4 at each time point). Data are presented as mean ± SE. Data were evaluated by one-way analysis of variance (ANOVA) followed by Scheffe’s test. A *P* value of less than 0.05 was considered statistically significant. * *P* < 0.05 compared to C group; # *P* < 0.05.

To determine the effect of IFX on osteocytic sclerostin expression in type 1 diabetes with periodontitis, we counted the number of sclerostin-positive osteocytes (Fig 4A) and measured sclerostin mRNA expression in alveolar bone of the distal area (Fig 4B). On day 3, the number of sclerostin-positive osteocytes in P or DP group was significantly higher than that in C group. However, the number of sclerostin-positive osteocytes in DP+IFX group was markedly lower than that in DP or P group. On day 20, the high number of sclerostin-positive osteocytes in DP group compared to that in C group was maintained whereas DP+IFX and P groups showed fewer sclerostin-positive osteocytes than the DP group. On day 3, sclerostin mRNA expression in DP+IFX group was lower than that in P or DP group. On day 20, we observed no significant difference in sclerostin mRNA expression level among these groups.

## Discussion

The precursor of TNF-α, a membrane-bound form, is expressed on the cell surface of activated macrophages and lymphocytes [33]. The membrane form of TNF-α is cleaved and then released as soluble form of TNF-α. IFX is a mouse-human chimeric anti-human TNF-α antibody that inhibits the binding of soluble TNF-α to TNF receptors. In previous studies using a TNF-α inhibitor, it has been demonstrated that TNF-α contributes to microvascular cell loss in diabetic retinopathy [34]. TNF-α also accelerates cartilage loss during fracture healing in type 1 diabetes rats or mice [35–38]. These findings indicate the TNF-α plays a crucial role in various complications of type 1 diabetes. In previous studies, type 1 diabetes rats with periodontitis have shown higher TNF-α expression and alveolar bone loss than rats with periodontitis [22, 26]. Our results showed that IFX, a TNF-α antagonist, decreased alveolar bone loss and expression levels of osteocytic RANKL and sclerostin in STZ-induced diabetes rats with periodontitis. This suggests that TNF-α is associated with alveolar bone loss in type 1 diabetes with periodontitis.

Alveolar bone loss is caused by bone resorption and osteoclasts induce bone resorption. In the present study, DP+IFX group showed lower number of cathepsin K-positive multinucleated cells compared to DP group on days 3 and 20. Our data indicate that TNF-α is involved in osteoclast formation in type 1 diabetes with periodontitis. The attenuation of osteoclast formation by the TNF inhibitor has also been shown during the healing process of femoral fracture in type 1 diabetes mice [35]. Pacios et al have recently reported that mice with periodontal infection show more RANKL-positive osteocytes and osteoblasts than non-infected mice, demonstrating that these cells contribute to osteoclast formation in periodontitis [6]. In addition, TNF-α has stimulatory effect on RANKL expression in osteocyte-like MLO-Y4 cells. Interestingly, we found a lower number of RANKL-positive osteocytes in DP+IFX group than that in DP group. This suggests that TNF-α is involved in osteocytic RANKL expression in type 1 diabetes with periodontitis.

In addition to bone resorption, alveolar bone loss is also affected by bone formation. We found that in the DP+IFX group had more bone formation on days 3 and 20 than the DP group. Interestingly, the DP+IFX group showed lower numbers of sclerostin-positive osteocytes on days 3 and 20 compared to DP group. It has been reported that TNF-α acts as an activator for sclerostin expression [29]. Additionally, serum sclerostin levels are higher in type 1 diabetes mice than those in normal mice [39]. One study has quantified sclerostin and TNF-α expression levels in MLO-Y4 osteocyte-like cells under high glucose conditions [40]. MLO-Y4 osteocyte-like cells do not express appreciable levels of sclerostin under basal conditions [41]. However, they can be induced to express sclerostin under conditions of high-glucose or exposure to reactive oxygen species [40]. H_2_O_2_-treated MLO-Y4 osteocyte-like cells also showed high TNF-α and sclerostin expression compared to non-treated cells. Antioxidant treatment and TNF-α knockdown attenuate high glucose-induced sclerostin expression, suggesting that hyperglycemia induces high sclerostin expression via enhancing the production of reactive oxygen species and TNF-α. Therefore, the attenuation of sclerostin-positive osteocytes in IFX treated rats implicates the stimulatory effect of TNF-α on osteocytic sclerostin expression in type 1 diabetes with periodontitis.

This study has some limitations. First, alveolar bone loss and bone formation were not measured using micro-CT and/or biomechanical measurements. Such measurements are needed for more clear confirmation of alveolar bone loss and bone formation. In previous studies, it has been demonstrated that TNF-α may also mediate osteoclast formation via stimulating RANKL expression in osteoblasts and periodontal ligament cells and extravasation of lymphocytes that can express RANKL [6, 27, 42–44]. Second, involvement of those cells in periodontal tissue as source of RANKL could not be excluded in TNF-α-mediated osteoclast formation of the type 1 diabetes with periodontitis. It is known that insulin/insulin like growth factor can stimulate the differentiation of osteoblasts [10, 45]. Deficiency of insulin/insulin like growth factor with anabolic activity on bone is associated with type 1 diabetes [10–12]. Besides TNF-α-mediated mechanism in the current study, decreased osteoblastogenesis by insulin/insulin like growth factor might also suppress bone formation in the type 1 diabetes with periodontitis. Third, this needs to be considered in further studies.

In summary, we found that treatment with IFX, a TNF-α antagonist, could attenuate alveolar bone loss, osteoclast formation, and RANKL-positive osteocytes in STZ-induced diabetes rats with periodontitis. In addition, the IFX treatment induced high osteoid formation and low sclerostin-positive osteocytes in STZ-induced diabetes rats with periodontitis. Our study suggests that TNF-α might mediate osteocytic RANKL and the sclerostin expression in type 1 diabetes with periodontitis.

## Supporting information

S1 Fig Experimental scheme for IFX treatment, body weight, and glucose levels during the experimental period. Experimental scheme (A). Body weight (B) and fasting glucose levels (C) for experimental groups. There are 6, 6, 8, and 8 rats in C, P, DP, and DP+IFX groups, respectively, at each time point.* *P* < 0.05 compared to C group. # *P* < 0.05.
(TIF)

S1 Table List of primer sequences used in this study. (DOCX)
