# Supplementary figures and images for: Tumor necrosis factor-α antagonist diminishes osteocytic RANKL and sclerostin expression in diabetes rats with periodontitis
Source: PLoS One. 2017 Dec 14;12(12):e0189702. doi: 10.1371/journal.pone.0189702 (PMC5730195; doi:10.1371/journal.pone.0189702)

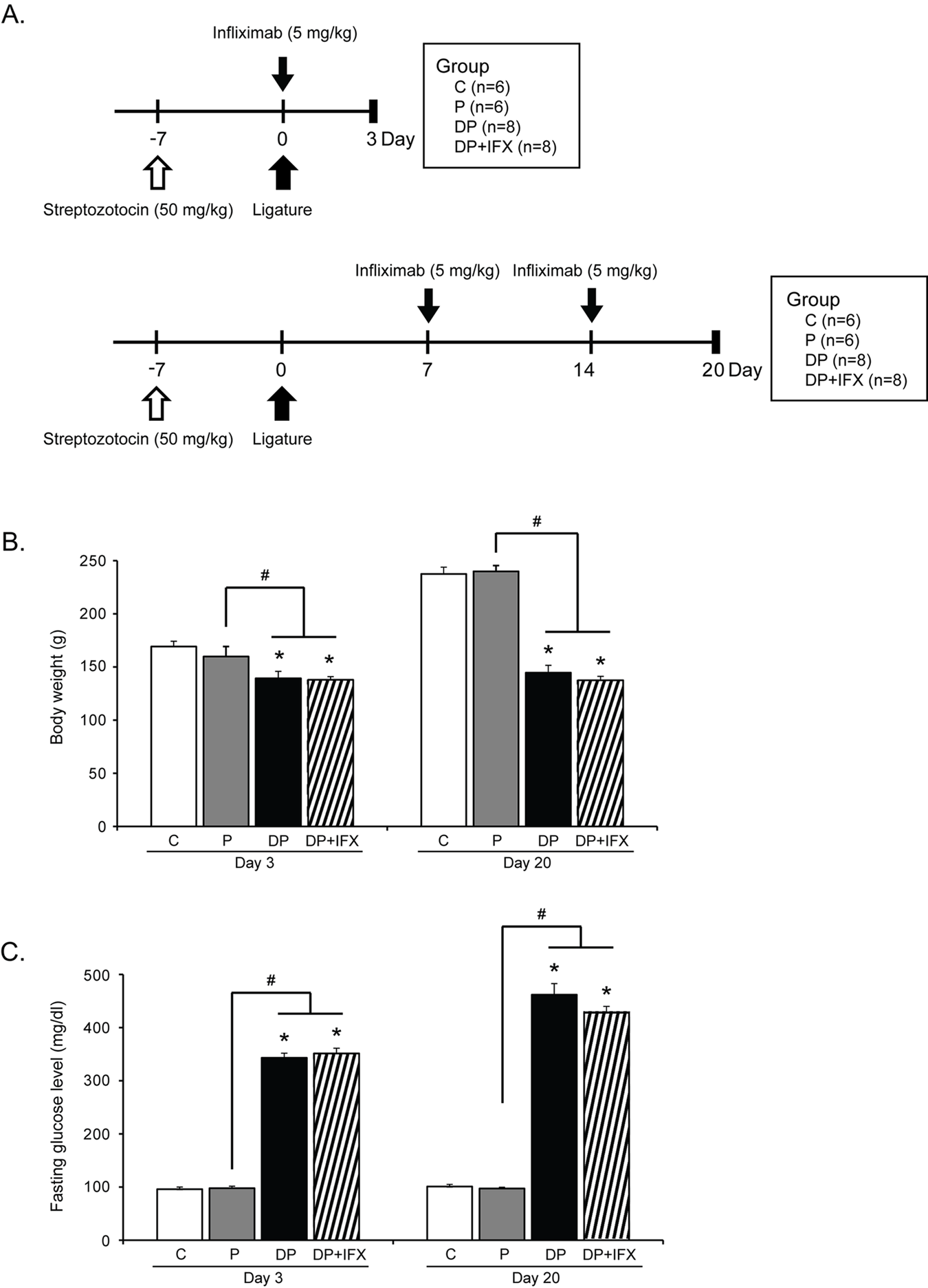

Supplement: S1 Fig — Experimental scheme (A). Body weight (B) and fasting glucose levels (C) for experimental groups. There are 6, 6, 8, and 8 rats in C, P, DP, and DP+IFX groups, respectively, at each time point.* P < 0.05 compared to C group. # P < 0.05. (TIF) [file pone.0189702.s001.tif]
