# Supplementary material for: Tumor necrosis factor-α antagonist diminishes osteocytic RANKL and sclerostin expression in diabetes rats with periodontitis
Source: PLoS One. 2017 Dec 14;12(12):e0189702. doi: 10.1371/journal.pone.0189702 (PMC5730195; doi:10.1371/journal.pone.0189702)
Supplement: S1 Table — (DOCX) [file pone.0189702.s002.docx]

**S1 Table.**

| **Gene** | **Sequence (5’ → 3’)** | |
| --- | --- | --- |
| IL-1β | F | CACCTCTCAAGCAGAGCACAG |
|  | R | GGTTCCATGGTGAAGTCAAC |
| Sclerostin | F | GAGAACAACCAGACCATGAAC |
|  | R | GCTCGCGGCAGCTGTACT |
| GAPDH | F | TTCTAGAGACAGCCGCATCT |
|  | R | TGGTAACCAGGCGTCCGATA |
